# Cancer Characteristic Gene Selection via Sample Learning Based on Deep Sparse Filtering

**DOI:** 10.1038/s41598-018-26666-0

**Published:** 2018-05-29

**Authors:** Jian Liu, Yuhu Cheng, Xuesong Wang, Lin Zhang, Z. Jane Wang

**Affiliations:** 1 School of Information and Control Engineering, China University of Mining and Technology, Xuzhou, 221116 China; 2 Electrical and Computer Engineering Department, University of British Columbia, V6T 1Z4 Vancouver, BC Canada

## Abstract

Identification of characteristic genes associated with specific biological processes of different cancers could provide insights into the underlying cancer genetics and cancer prognostic assessment. It is of critical importance to select such characteristic genes effectively. In this paper, a novel unsupervised characteristic gene selection method based on sample learning and sparse filtering, Sample Learning based on Deep Sparse Filtering (SLDSF), is proposed. With sample learning, the proposed SLDSF can better represent the gene expression level by the transformed sample space. Most unsupervised characteristic gene selection methods did not consider deep structures, while a multilayer structure may learn more meaningful representations than a single layer, therefore deep sparse filtering is investigated here to implement sample learning in the proposed SLDSF. Experimental studies on several microarray and RNA-Seq datasets demonstrate that the proposed SLDSF is more effective than several representative characteristic gene selection methods (e.g., RGNMF, GNMF, RPCA and PMD) for selecting cancer characteristic genes.

## Introduction

Cancer is related to abnormal cell growth which has the potential to invade or spread to other parts of the human body. Currently there are more than 100 types of known cancers that are very detrimental for humans. According to the World Health Organization’s World Cancer Report 2014, about 14.1 million new cases of cancer emerged globally (excluding non-melanoma skin cancer). It caused about 8.2 million deaths, accounting for 14.6% of all human deaths^1^. In the United States, the average five-year survival rate for cancer is 66%^2^. Genetically, genes that regulate cell growth and differentiation could be altered to develop a normal cell into a cancer cell. These genes can usually be divided into two broad categories: oncogenes which promote cell growth and reproduction, and suppressor genes which inhibit cell division and survival^3^. In contemporary molecular biology, it remains a challenge to accurately identify such genes relevant to key cellular processes.

The advances of DNA microarray and deep sequencing technologies have made it possible for biologists to measure expression levels of thousands of genes simultaneously^4,5^. These genes can be detected more comprehensively and more detailed than ever before. However, in each gene expression dataset, the number of genes is so huge (thousands or even more than 10,000) that it is extremely difficult to analyze the whole set of gene expression data. Fortunately, for an exact biological process, only a small set of genes may take part in the regulation of gene expression level^6,7^. Such a small set of genes usually are referred as characteristic genes. Identification of the characteristic genes associated with special biological processes of different types of cancers could provide important insights into the underlying genetics and prognostic assessment of cancer. Therefore, effective identification of such characteristic genes has been an important research topic, which technically is closely related to feature selection.

Recently, deep learning, originally proposed by Hinton *et al*.^8^ to learn a multiple hierarchical network by training^9^, has drawn increasing attention. With the obtained deep non-linear network, deep learning can provide a complex function approximation. Numerous deep learning methods were proposed for different learning tasks, such as feature learning, classification, and recognition. The most commonly used models include deep belief networks (DBNs)^8^, stacked auto-encoders (SAEs)^10^, and convolutional neural networks (CNNs)^11^. These models have been successfully applied to numerous fields (e.g., image processing, natural language processing, and medical data analytics) and achieved promising performances. Particularly, they have been used to analyze gene expression data. For example, SAE was successfully applied to enhance cancer diagnosis and classification based on gene expression data by Fakoor *et al*.^12^. Liu *et al*. proposed the sample expansion based 1-dimensional CNN for classifying tumor gene expression data^13^. However, training DBN, SAE and CNN models is often time-consuming and labor expensive, since a large number of hyperparameters need to be tuned. Sparse filtering, an unsupervised feature learning algorithm, works by optimizing the sparsity of the feature distribution and it is essentially hyperparameter-free. Since the critical idea of sparse filtering is to avoid explicit modeling of the data distribution, this can give rise to a simple formulation and permits learning effectively. Furthermore, sparse filtering can be extended into multi-layer networks. Deep sparse filtering can be used to learn meaningful features in additional layers by using greedy layer-wise stacking^14^. Therefore, in this paper, we employ deep sparse filtering to select characteristic genes.

Several deep learning methods have been explored to select cancer genes. Danaee *et al*. used stacked denoising autoencoder (SDAE) to detect breast cancer and identify relevant genes^15^. In their work, firstly, SDAE is used to extract functional features from gene expression profiles. Then, the performance of the extracted representation is evaluated through supervised classification models. Lastly, a set of highly interactive genes are identified by analyzing the SDAE connectivity matrices. Ibrahim *et al*. selected multi-level gene/miRNA by using DBN and active learning to enhance the classification accuracy^16^. The major steps of the approach are described as follows: (1) Use DBN to extract high level representations of the gene expression profiles; (2) Apply a feature selection method to rank genes; (3) Obtain the finally selected genes using active learning. Both SDAE^15^ and DBN^16^ are supervised methods, and can learn high level features of the gene expression data. Feature learning maps a high-dimensional feature space of the original data into a low-dimensional space so that the data can be better represented by the transformed feature space. Since each feature in the gene expression data represents a gene, if we employ traditional feature learning methods, the original feature space will be changed and we cannot specify the exact genes in the new feature space. Therefore traditional feature learning is not applicable to characteristic gene selection. In addition, since gene expression datasets generally are with high dimensional features and small sample size, SDAE and DBN suffer from serious overfitting when applied to gene expression data. Moreover, SDAE and DBN perform poorly when the unlabeled data is abundant while the labeled data is scarce, which is exactly our case. Considering the limited labelled data in our problem, unsupervised learning is more suitable.

To address the above concerns, different from previous feature learning methods, we propose the idea of sample learning, an unsupervised method, for selecting genes with deep learning models. Sample learning transforms the sample space of gene expression data and ensures that the features (or genes) can be better represented by the transformed sample space so that we can specify the exact characteristic genes from the transformed sample space.

In this paper, by combining sample learning and deep sparse filter, a novel unsupervised characteristic gene selection method, which is named as Sample Learning based on Deep Sparse Filtering (SLDSF), is proposed for cancer characteristic gene selection. In the proposed method, firstly, the idea of sample learning for selecting characteristic genes is presented. Then the applicability of sample learning using sparse filtering is explained. Finally, the deep sparse filtering framework is extended by using the feed-forward network. Our later tests on gene expression datasets demonstrate that cancer characteristic genes can be effectively selected using the proposed SLDSF.

The remainder of the paper is structured as follows. In Section 2, the proposed SLDSF for selecting cancer characteristic genes is presented. When compared the proposed SLDSF with four unsupervised methods: RGNMF, GNMF, RPCA and PMD, experimental results on several cancer gene expression datasets are reported in Section 3. In Section 4, the conclusions are given.

## Methods

### Sparse Filtering

Sparse filtering^14^, an unsupervised feature learning method, is easy to implement with only one hyperparameter. It optimizes the sparsity of the feature distribution. The main idea of sparse filtering is to avoid explicit modeling of the data distribution by a simple formulation and thus permits effective learning.

Denote a gene expression dataset as ${\boldsymbol{A}}\in {{\mathbb{R}}}^{m\times n}$, where each row represents a feature and each column represents a sample. Denote ${\boldsymbol{F}}\in {{\mathbb{R}}}^{d\times n}$ as the feature distribution matrix over ***A***. The entry ***F***_*ij*_ in ***F*** represents the activity of the *i*-th feature on the *j*-th sample. By imposing sparse constraints on ***F***, a matrix ${\boldsymbol{W}}\in {{\mathbb{R}}}^{m\times d}$ can be obtained which satisfies ${\boldsymbol{F}}={{\boldsymbol{W}}}^{{\rm{T}}}{\boldsymbol{A}}$. And each column in ***W*** can be viewed as a sparse filter. Sparse filtering involves three steps: normalizing ***F*** by rows, then normalizing ***F*** by columns and finally summing up the absolute values of all elements. Denote ${{\boldsymbol{F}}}_{in}\in {{\mathbb{R}}}^{1\times n}(i=1,2,3,\cdots ,d)$ as the *i*-th row of ***F*** and ${{\boldsymbol{F}}}_{dj}\in {{\mathbb{R}}}^{d\times 1}(j=1,2,3,\cdots ,n)$ as the *j*-th column of ***F***. To be specific, each feature of ***F*** is divided by the *L*_2_-norm across all samples: ${\tilde{{\boldsymbol{F}}}}_{in}={{{\boldsymbol{F}}}_{in}/\Vert {{\boldsymbol{F}}}_{in}\Vert }_{2}$,which normalizes each feature to be equally active. Then, each sample is divided by the *L*_2_-norm across all features: ${\hat{{\boldsymbol{F}}}}_{dj}={{\tilde{{\boldsymbol{F}}}}_{dj}/\Vert {\tilde{{\boldsymbol{F}}}}_{dj}\Vert }_{2}$ to make all samples lie on the unit *L*_2_-ball. Finally, all the normalized elements are optimized for sparseness by using the *L*_1_-norm. Therefore the objective function of sparse filtering can be expressed as follows:

1 $$\min \,\sum _{j=1}^{n}{\Vert {\hat{{\boldsymbol{F}}}}_{dj}\Vert }_{1}.$$

The sparse filtering is implemented by the L-BFGS method, a commonly used iterative algorithm for solving unconstrained nonlinear optimization problems^17^. In the objective function Eq. (1), the feature distribution has shown population sparsity, high dispersal as well as lifetime sparsity, which have been investigated in^18,19^.

#### Population sparsity

Population sparsity means that each sample should have a few active (non-zero) features. The term ${\Vert {\hat{{\boldsymbol{F}}}}_{dj}\Vert }_{1}$ in Eq. (1) reflects this characteristic of the features on the *j*-th sample. Because ${\hat{{\boldsymbol{F}}}}_{{dj}}$ is constrained to lie on the unit *L*_2_-ball, the objective function can be minimized when the features are sparse.

#### High dispersal

High dispersal means that the distribution should have similar statistics for different features. Specifically, the considered statistics are the mean squared activations of each feature by averaging the squared values in the feature matrix across the samples. For all features, the statistics should be roughly the same, suggesting that the contributions of all features should be roughly same. In the first step of sparse filtering, each feature of ***F*** is divided by the *L*_2_-norm across all samples, ${\tilde{{\boldsymbol{F}}}}_{in}={{{\boldsymbol{F}}}_{in}/\Vert {{\boldsymbol{F}}}_{in}\Vert }_{2}$, to normalize each feature to be equally active.

#### Lifetime sparsity

Lifetime sparsity means each feature should be active in a few samples, which ensures that the features should be discriminative enough to distinguish samples. Concretely, a few active (non-zero) elements should be included in each row of the feature distribution matrix. In the objective function of sparse filtering, the characteristic of lifetime sparsity is guaranteed by population sparsity and high dispersal. Due to the population sparsity, many non-zero elements can be obtained in the feature distribution matrix. These zero elements are roughly evenly distributed across all features due to high dispersal. Accordingly, each feature would have a great number of non-zero elements and thus be lifetime sparse.

### Sample Learning for Characteristic Gene Selection

Traditionally, feature learning algorithms usually transform the feature space to achieve dimensionality reduction. To be more specific, a high-dimensional feature space of the original data is mapped into a low-dimensional feature space by using feature learning methods which maintain the distance information between samples. In other words, feature learning is a process of representing the samples in the low dimensional feature space which is obtained by using some mapping or rescaling methods. Feature learning can be used for classification tasks by transforming the feature space to achieve the desired results.

**Figure 1:**
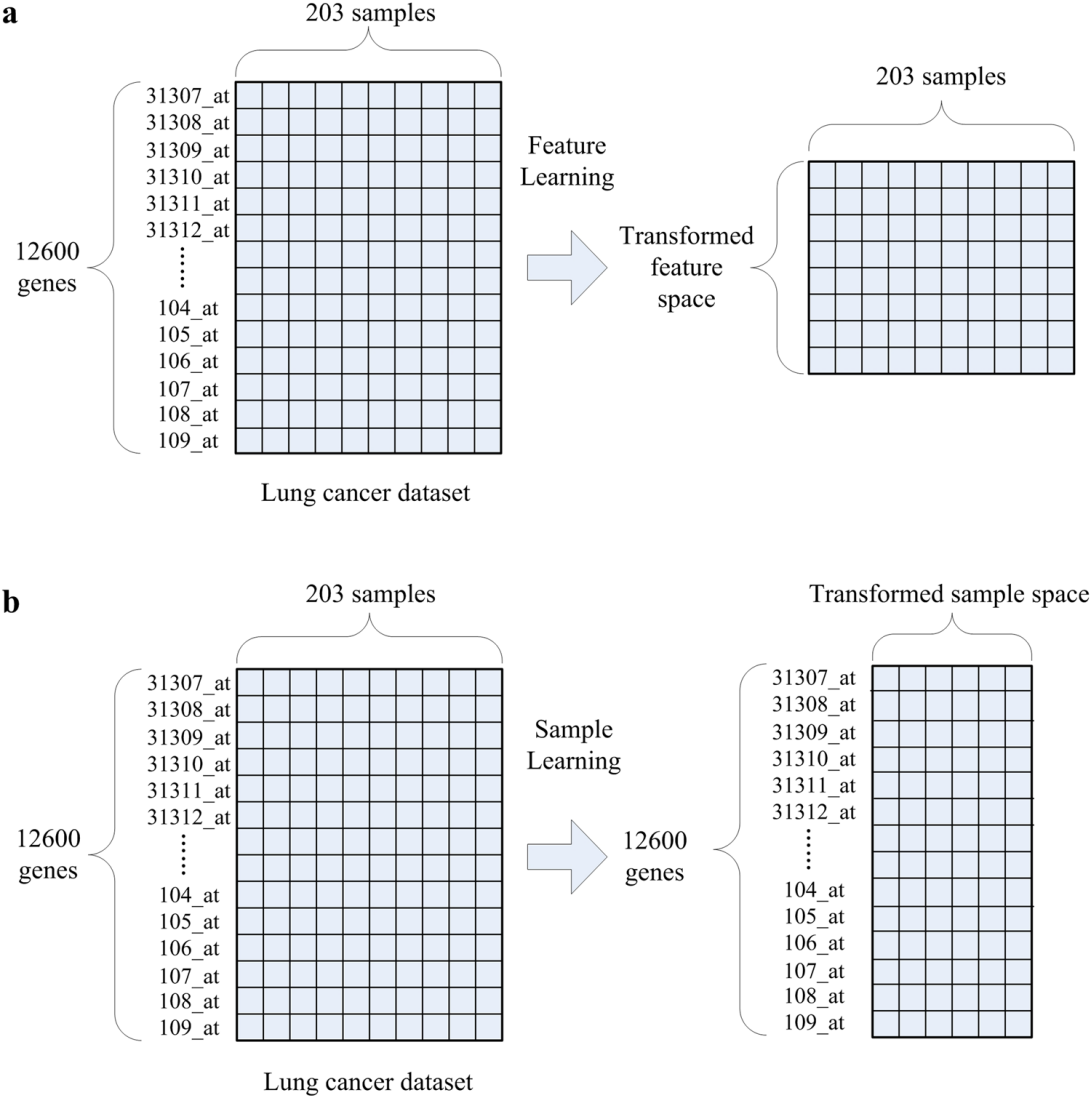
The differences between sample learning and feature learning. (**a**) A feature learning model for the lung cancer dataset. (**b**) A sample learning model for the lung cancer dataset.

However, direct feature learning is not applicable for characteristic gene selection. In our problem, since each feature represents a gene, if we use feature learning methods to process the gene expression data, the original feature space will be changed and we cannot identify the exact genes in the new feature space. In order to explain this problem intuitively, a common feature learning model is shown in Fig. 1(a).

The lung cancer dataset, which contains 12600 genes on 203 samples, is taken as an example, where each row represents a gene (some names of genes are provided in Fig. 1(a)) and each column represents a sample. After being processed by feature learning methods, the feature space of the lung cancer dataset is changed and we cannot locate the exact genes in the transformed feature space. In this paper, our goal is to find a group of characteristic genes associated with special biological processes of different cancers which may illuminate the underlying genetics and contribute to the prognostic assessment. Obviously, without knowing the exact genes in the transformed feature space, our goal cannot be achieved. Therefore, direct feature learning is not preferred for characteristic gene selection.

To address this problem, sample learning is proposed to analyze gene expression data in the proposed method. Compared to feature learning, sample learning transforms the sample space. The illustration of a sample learning model for the lung cancer dataset is shown in Fig. 1(b). After being processed by sample learning, the feature space of the lung cancer dataset remains unchanged while the sample space is transformed. In this case, the information of each gene can be better represented by the transformed sample space. Then we can select characteristic genes through some feature selection strategies from the processed matrix in Fig. 1(b). In short, sample learning is a process that the features are represented by a transformed sample space which is obtained via some mapping or rescaling algorithms.

### Applicability Analysis of Sample Learning Using Sparse Filtering

In the subsection above, the idea of sample learning was introduced for cancer characteristic gene selection. Particularly, we adopt sparse filtering for sample learning. As mentioned above, the feature learning objective function in Eq. (1) makes the feature distribution have three desirable characteristics. Similarly, sample learning also provides these characteristics of the sample distribution.

Suppose there is a sample distribution matrix over a gene expression dataset, where each row is a sample, each column is a gene, and the elements are the activities of samples on specific genes. A detailed explanation of how sample learning satisfies the three desirable characteristics of the sample distribution is as follows:

#### Population sparsity

Population sparsity requires that each gene should have a few non-zero samples. Specifically, for each gene (one column) in the sample distribution matrix, only a small number of non-zero entries are required. These non-zero entries represent this gene is differentially expressed on the non-zero samples. This indicates that one gene is usually impossible differentially expressed on all samples. The cancer characteristic genes can be selected according to these differentially expressed genes.

#### Lifetime sparsity

Lifetime sparsity requires each sample should be active on a few genes which ensure that the samples should be discriminative enough to distinguish genes. In a gene expression dataset, each sample has the expression levels of all genes, but only a small number of genes are differentially expressed on each sample. Since our purpose is to select differentially expressed genes, the samples are discriminative enough to distinguish genes. Here, the non-zero entries in each sample can be represented as the differentially expressed genes and the zero entries are represented as the non-differentially expressed genes. Therefore, each sample in the sample distribution matrix should allow limited non-zero entries.

#### High dispersal

High dispersal requires that the distribution should have similar statistics on different samples which suggest that the contributions of all samples should be roughly same. This property prevents the same samples are always active and guarantees the extracted samples keep orthogonal^19^. After sample learning by enforcing high dispersal, the extracted samples can more effectively represent the differential expression levels of genes and are conducive to select characteristic genes.

### The Framework of SLDSF

In this subsection, firstly, the Sample Learning based Sparse Filtering (SLSF) method is presented. Then the SLSF method is expanded into SLDSF, a deep structure for learning more meaningful representations^14^.

Denote a gene expression dataset as ${\boldsymbol{B}}\in {{\mathbb{R}}}^{n\times m}$, where each row means a sample and each column means a gene. In order to eliminate the dimensional effect between indicators, the gene expression dataset is normalized into ***X*** which is used to implement sample learning. Denote a sample distribution matrix over ***X*** as ${\boldsymbol{S}}\in {{\mathbb{R}}}^{t\times m}$. The element ***S***_*ij*_ in ***S*** is the activity of the *i*-th sample on the *j*-th gene. A sparse filter matrix ${\boldsymbol{Y}}\in {{\mathbb{R}}}^{n\times t}$ which satisfies the soft-absolute function ${\boldsymbol{S}}=\sqrt{{({{\boldsymbol{Y}}}^{{\rm{T}}}{\boldsymbol{X}})}^{2}+{10}^{-8}}$ can be obtained. Each column in ***Y*** can be regarded as a sparse filter. Denote ${{\boldsymbol{S}}}_{im}\in {{\mathbb{R}}}^{1\times m}(i=1,2,3,\cdots ,t)$ as the *i*-th row of ***S*** and ${{\boldsymbol{S}}}_{tj}\in {{\mathbb{R}}}^{t\times 1}(j=1,2,3,\cdots ,m)$ as the *j*-th column of ***S***. Similar to sparse filtering, the sample learning based sparse filtering also has three steps: normalizing ***S*** by rows with the *L*_2_-norm: ${\tilde{{\boldsymbol{S}}}}_{im}={{{\boldsymbol{S}}}_{im}/\Vert {{\boldsymbol{S}}}_{im}\Vert }_{2}$, then normalizing ${\tilde{{\boldsymbol{S}}}}_{im}$ by columns with the *L*_2_-norm: ${\hat{{\boldsymbol{S}}}}_{tj}={{\tilde{{\boldsymbol{S}}}}_{tj}/\Vert {\tilde{{\boldsymbol{S}}}}_{tj}\Vert }_{2}$ and finally all the normalized elements are optimized for sparseness by using the *L*_1_-norm: $\sum _{j=1}^{m}{\Vert {\hat{{\boldsymbol{S}}}}_{tj}\Vert }_{1}$. For *m* features in the gene expression dataset ***B***, the objective of the SLSF method can be written as

2 $$\min \,\sum _{j=1}^{m}{\Vert {\hat{{\boldsymbol{S}}}}_{tj}\Vert }_{1}$$

**Figure 2:**
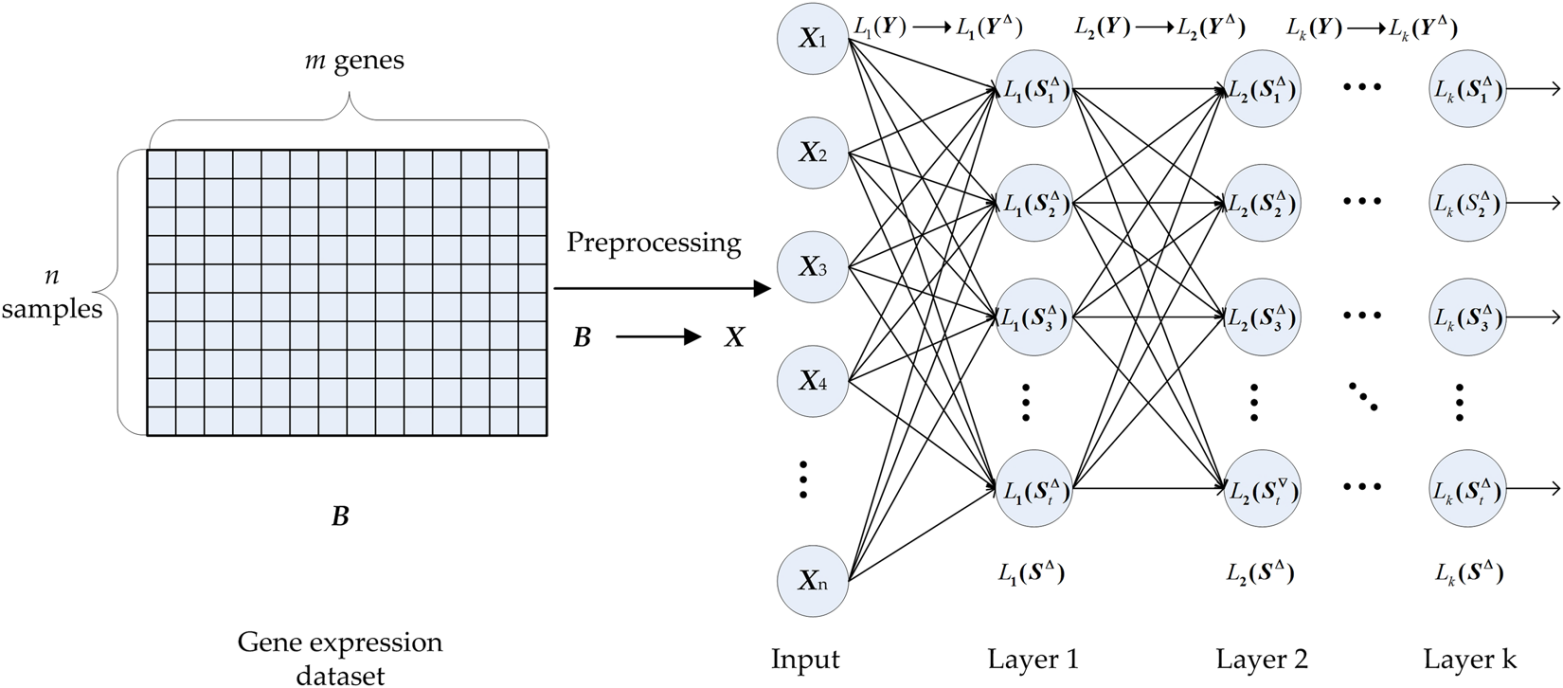
The framework of sample learning with SLDSF on gene expression data.

SLSF can also be implemented by the L-BFGS method. The SLSF method can be regarded as the first layer of the SLDSF method. After training a single layer of samples with SLSF, one can compute the normalized samples and then use these as the input to SLDSF for learning the second layer of samples. The rest multiple layers can be learnt in the same manner. The framework of sample learning with SLDSF on gene expression data is described in Fig. 2.

Firstly, the gene expression dataset is preprocessed by the following formula

3 $${\boldsymbol{X}}=({\boldsymbol{B}}-mean({\boldsymbol{B}}))\frac{std({\boldsymbol{X}})}{std({\boldsymbol{B}})}+mean({\boldsymbol{X}}),$$

where *mean*(***B***) is the mean of gene expression data matrix ***B*** by row, *std*(***B***) is the standard deviation of gene expression data matrix (***B***) by row, *std*(***X***) is the standard deviation of the expected matrix ***X*** by row and *mean*(***X***) is the mean of the expected matrix ***X*** by row. Here, *std*(***X***) and *mean*(***X***) are simply set to be 1 and 0 respectively.

Secondly, the preprocessed matrix ***X*** in Eq. (3) is regarded as the input layer to implement sample learning with SLDSF. In Fig. 2, suppose we need *k* layers in SLDSF, in addition to the input layer. We denote ***X***_*n*_ as the input layer which has *n* samples to be learned, ${L}_{k}({{\boldsymbol{S}}}^{{\rm{\Delta }}})$ as the output matrix of the *k*-th layer, ${L}_{k}({{\boldsymbol{S}}}_{t}^{{\rm{\Delta }}})$ as the *t*-th sample in the output matrix ${L}_{k}({{\boldsymbol{S}}}^{{\rm{\Delta }}})$, *L*_*k*_(***Y***) as the sparse filter matrix of the of the *k*-th layer and ${L}_{k}({{\boldsymbol{Y}}}^{{\rm{\Delta }}})$ as the optimal sparse filter matrix of the *k*-th layer.

For Layer 1 in Fig. 2, the SLSF can be taken as the Layer 1 of SLDSF. Here, we denote ${L}_{1}({\boldsymbol{S}})=\sqrt{{({L}_{1}{({\boldsymbol{Y}})}^{{\rm{T}}}{\boldsymbol{X}})}^{2}+{10}^{-8}}$ as the sample distribution matrix of Layer 1, the objective function in Layer 1 can be written as *L*_1_(*J*), then we have

4 $$min\,{L}_{1}(J)=\sum _{j=1}^{m}{\parallel {L}_{1}({\hat{{\boldsymbol{S}}}}_{tj})\parallel }_{1}={\sum _{j=1}^{m}\parallel \frac{{L}_{1}({\mathop{{\boldsymbol{S}}}\limits^{ \sim }}_{tj})}{{\parallel {L}_{1}({\mathop{{\boldsymbol{S}}}\limits^{ \sim }}_{tj})\parallel }_{2}}\parallel }_{1},$$

where ${L}_{1}({\hat{{\boldsymbol{S}}}}_{tj})$ is the normalized matrix by normalizing ${L}_{1}(\tilde{{\boldsymbol{S}}})$ via columns with the *L*_2_-norm: ${L}_{1}({\hat{{\boldsymbol{S}}}}_{tj})={L}_{1}({\tilde{{\boldsymbol{S}}}}_{tj})/{\Vert {L}_{1}({\tilde{{\boldsymbol{S}}}}_{tj})\Vert }_{2}$, and ${L}_{1}(\tilde{{\boldsymbol{S}}})$ is the normalized matrix by normalizing *L*_1_(***S***) via rows with the *L*_2_-norm: ${L}_{1}({\tilde{{\boldsymbol{S}}}}_{im})={L}_{1}({{\boldsymbol{S}}}_{im})/{\Vert {L}_{1}({{\boldsymbol{S}}}_{im})\Vert }_{2}$.

In order to obtain the optimal solution of Eq. (4), we use the Back Propagation (BP) method to adjust the sparse filter matrix *L*_1_(***Y***). The gradient of *L*_1_(***Y***) on the objective function *L*_1_(*J*) in Eq. (4) can be written as

5 $${\nabla }_{{L}_{1}({\boldsymbol{Y}})}{L}_{1}(J)({L}_{1}({\boldsymbol{Y}}))=\frac{\partial {L}_{1}(J)}{\partial {L}_{1}({\boldsymbol{Y}})}.$$

With the chain rule, Eq. (5) can be expanded into the following form

6 $${L}_{1}({\rm{\Delta }}{\boldsymbol{Y}})={\nabla }_{{L}_{1}({\boldsymbol{Y}})}{L}_{1}(J)({L}_{1}({\boldsymbol{Y}}))=\frac{\partial {L}_{1}(J)}{\partial {L}_{1}({\boldsymbol{Y}})},$$

where ${L}_{1}({\rm{\Delta }}{\boldsymbol{Y}})$ is the gradient of *L*_1_(***Y***) on *L*_1_(*J*) in Eq. (4). The objective function *L*_1_(*J*) and ${L}_{1}({\rm{\Delta }}{\boldsymbol{Y}})$ can be optimized by using the L-BFGS method^17^ to achieve the optimal sparse filter matrix ${L}_{1}({{\boldsymbol{Y}}}^{{\rm{\Delta }}})$. The output matrix ${L}_{1}({{\boldsymbol{S}}}^{{\rm{\Delta }}})$ of Layer 1 is obtained by using ${L}_{1}({{\boldsymbol{Y}}}^{{\rm{\Delta }}})$

7 $${L}_{1}({{\boldsymbol{S}}}^{{\rm{\Delta }}})=\sqrt{{({L}_{1}{({{\boldsymbol{Y}}}^{{\rm{\Delta }}})}^{{\rm{T}}}{\boldsymbol{X}})}^{2}+{10}^{-8}}.$$

After training the samples of Layer 1 in SLDSF, the optimal sample distribution matrix ${L}_{1}({{\boldsymbol{S}}}^{{\rm{\Delta }}})$ is obtained as the output of Layer 1.

For Layer 2, we choose the feedforward network to train the samples. In Layer 2, we firstly normalize ${L}_{1}({{\boldsymbol{S}}}^{{\rm{\Delta }}})$ by rows, and then by columns using the *L*_2_-norm. The normalized ${L}_{1}({{\boldsymbol{S}}}^{{\rm{\Delta }}})$ is taken as the input to SLDSF for learning the second layer of samples. With the computation process of Layer 1, we can obtain the optimal sparse filter matrix ${L}_{2}({{\boldsymbol{Y}}}^{{\rm{\Delta }}})$ and the output sample distribution matrix ${L}_{2}({{\boldsymbol{S}}}^{{\rm{\Delta }}})$ of Layer 2. The rest multiple layers can be learnt in the same manner. Finally, we can obtain the final output sample distribution matrix ${L}_{k}({{\boldsymbol{S}}}^{{\rm{\Delta }}})$ in Layer *k*. Note that, since SLDSF randomly initializes the sparse filter matrix, the results from running the SLDSF algorithm multiple times will not be exactly the same. The cancer characteristic genes are selected according to ${L}_{k}({{\boldsymbol{S}}}^{{\rm{\Delta }}})$, and the detail ideas are presented in the following subsection.

To summarize, the major steps of the proposed SLDSF algorithm are described in Table 1.

**Table 1:** The SLDSF algorithm.

Input: Gene expression dataset: ***B***. The number of samples needs to be learned: *t*. The number of layers: ***k***. Output: Optimal sample distribution matrix ${L}_{k}({{\boldsymbol{S}}}^{{\rm{\Delta }}})$.
Initialize ${L}_{1}({\boldsymbol{Y}})$, ${L}_{2}({\boldsymbol{Y}})$, $\cdots$, ${L}_{k}({\boldsymbol{Y}})$ Normalize gene expression dataset ${\boldsymbol{B}}$ by Eq. (3) as the input of Layer 1. for $i=1$; $i\le k$; *i*++ Obtain ${L}_{i}(J)$ by Eq. (4) Calculate ${L}_{1}({\rm{\Delta }}{\boldsymbol{Y}})$ by Eq. (6) Update ${L}_{i}({{\boldsymbol{Y}}}^{{\rm{\Delta }}})$ by L-BFGS method until convergence Obtain ${L}_{i}({{\boldsymbol{S}}}^{{\rm{\Delta }}})$ by Eq. (7) Normalize ${L}_{i}({{\boldsymbol{S}}}^{{\rm{\Delta }}})$ by ${L}_{2}$-norm as the input of Layer *i* + 1 end for Output ${L}_{k}({{\boldsymbol{S}}}^{{\rm{\Delta }}})$

### Cancer Characteristic Gene Selection by SLDSF

After being processed by SLDSF, the gene expression dataset can be better represented by the optimal sample distribution matrix ${L}_{k}({{\boldsymbol{S}}}^{{\rm{\Delta }}})$ since ${L}_{k}({{\boldsymbol{S}}}^{{\rm{\Delta }}})$ contains the desirable properties of the sample distribution. Therefore, cancer characteristic genes can be selected by exploring ${L}_{k}({{\boldsymbol{S}}}^{{\rm{\Delta }}})$ effectively. The main idea is explained as follows.

The optimal sample distribution matrix ${L}_{k}({{\boldsymbol{S}}}^{{\rm{\Delta }}})$ can be described as

8 $${L}_{k}({{\boldsymbol{S}}}^{{\rm{\Delta }}})=[\begin{array}{cccc}{s}_{11} & {s}_{12} & \cdots  & {s}_{1m}\\ {s}_{21} & {s}_{22} & \cdots  & {s}_{2m}\\ \vdots  & \vdots  & \ddots  & \vdots \\ {s}_{t1} & {s}_{t2} & \cdots  & {s}_{tm}\end{array}].$$

According to Eq. (7), all elements in ${L}_{k}({{\boldsymbol{S}}}^{{\rm{\Delta }}})$ are non-negative. Then, we sum the elements by columns to obtain the evaluating vector

9 $${L}_{k}({\hat{{\boldsymbol{S}}}}^{{\rm{\Delta }}})=[\begin{array}{cccc}\sum _{t=1}^{t}|{{\boldsymbol{S}}}_{t1}| & \sum _{t=1}^{t}|{{\boldsymbol{S}}}_{t2}| & \cdots  & \sum _{t=1}^{t}|{{\boldsymbol{S}}}_{tm}|\end{array}].$$

Generally, the more differentially expressed the gene is, the larger the corresponding element in ${L}_{k}({\hat{{\boldsymbol{S}}}}^{{\rm{\Delta }}})$ is. Hence, we can sort the items of ${L}_{k}({\hat{{\boldsymbol{S}}}}^{{\rm{\Delta }}})$ in a descending order, and then take the top *h* genes as the characteristic ones.

## Results and Discussion

This section reports several experimental results. We first test the proposed method on three publicly available microarray datasets, i.e., lung cancer dataset^20^, leukemia dataset^21^ and diffuse large B cell lymphoma (DLBCL) dataset^22^. We also test our method on two RNA-Seq datasets, i.e., esophageal cancer (ESCA) and squamous cell carcinoma of head and neck (HNSC). These five datasets are summarized in Table 2, and they can be found in Supplementary Datasets. To demonstrate the effectiveness of the proposed SLDSF method for selecting cancer characteristic genes, four commonly used gene selection methods: RGNMF^23^, GNMF^24^, RPCA^25^ and PMD^26^ are employed for comparison. The detailed method description can be found in Supplementary S1. We also provide the codes of all methods used in this paper in Supplementary Codes. In this paper, the programs were implemented by using Matlab2014a on a PC equipped with an Intel Core i5 and 8 GB memory.

**Table 2:** Summary of gene expression datasets.

Dataset		Name	Number of		
			Genes	Samples	Classes
Microarray	Lung Cancer	Lung adenocarcinomas, squamous cell lung carcinomas, pulmonary carcinoids, small-cell lung carcinomas cases, normal lung samples	12600	203	5
	Leukemia	Acute myelogenous leukemia, acute lymphoblastic leukemia	5000	38	2
	DLBCL	‘Cured’ patients, ‘fatal/refractory’ patients	7129	58	2
RNA-Seq	ESCA	Diseased samples, normal samples	20502	192	2
	HNSC	Diseased samples, normal samples	20502	418	2

### Gene Ontology Analysis

For fair comparisons, 100 genes were selected by SLDSF, RGNMF, GNMF, RPCA and PMD methods. The 100 genes selected by SLDSF can be found in Supplementary S2. The GO (Gene Ontology) enrichment of functional annotation of the selected characteristic genes by the five methods was detected by ToppFun which can be used to describe characteristic genes in the input or query set and to help discover what functions these genes may have in common^27^^,^^28^. The tool is publicly available at http://toppgene.cchmc.org/enrichment.jsp. In this paper, GO: Biological Process is the main objective to analysis.

### Test on Microarray Datasets

This subsection reports experimental results on three microarray datasets: lung cancer dataset, leukemia dataset and DLBCL dataset. SLDSF is a deep structure for sample learning. We first tested the influence of the number of layers and the number of samples. The results can be found in Supplementary S3. From Supplementary S3, the proposed SLDSF can obtain the best results on all three datasets when the numbers of layers and samples are 3 and 200, respectively. So we adopt the 3-Layer SLDSF with 200 samples in the later comparisons. The results of five methods on lung cancer dataset, leukemia dataset and DLBCL dataset were summarized in Tables 3, 4 and 5, respectively. In the tables, the best results among five methods were shown in bold. For simplicity, only the P-values of top 10 GO terms were shown in this paper.

**Table 3:** The P-Values of GO terms corresponding to different methods on the lung cancer dataset.

ID	Name	SLDSF	RGNMF	GNMF	RPCA	PMD
		P-Value	P-Value	P-Value	P-Value	P-Value
GO:0000184	nuclear-transcribed mRNA catabolic process, nonsense-mediated decay	**5**.**05E-72**	2.16E-16	3.16E-16	None	5.24E-15
GO:0006614	SRP-dependent cotranslational protein targeting to membrane	**7**.**03E-72**	2.77E-16	4.04E-16	None	6.58E-15
GO:0006613	cotranslational protein targeting to membrane	**1**.**69E-71**	4.47E-16	6.53E-16	None	1.02E-14
GO:0045047	protein targeting to ER	**9**.**22E-71**	7.09E-16	1.04E-15	None	1.56E-14
GO:0072599	establishment of protein localization to endoplasmic reticulum	**4**.**68E-70**	9.91E-16	1.45E-15	None	2.12E-14
GO:0070972	protein localization to endoplasmic reticulum	**4**.**61E-67**	5.15E-15	7.50E-15	None	9.63E-14
GO:0019080	viral gene expression	**5**.**18E-64**	3.47E-14	5.19E-14	None	4.49E-13
GO:0044033	multi-organism metabolic process	**4**.**62E-63**	6.77E-14	1.01E-13	None	1.01E-13
GO:0019083	viral transcription	**6**.**96E-63**	3.91E-13	5.66E-13	None	5.14E-12
GO:0006415	translational termination	**5**.**27E-62**	5.94E-15	8.91E-15	None	8.79E-14

**Table 4:** The P-Values of GO terms corresponding to different methods on the leukemia dataset.

ID	Name	SLDSF	RGNMF	GNMF	RPCA	PMD
		P-Value	P-Value	P-Value	P-Value	P-Value
GO:0006955	immune response	**2**.**69E-18**	4.14E-12	2.76E-11	3.45E-15	1.83E-11
GO:0001775	cell activation	8.94E-18	1.40E-14	1.35E-13	**5**.**14E-19**	8.60E-13
GO:0045321	leukocyte activation	**2**.**28E-16**	5.89E-13	5.34E-11	4.72E-16	4.01E-11
GO:0007159	leukocyte cell-cell adhesion	**5**.**86E-16**	3.56E-13	4.58E-15	6.05E-14	4.07E-11
GO:0046649	lymphocyte activation	**8**.**59E-16**	3.13E-12	2.63E-09	2.95E-15	2.43E-11
GO:0016337	single organismal cell-cell adhesion	**1**.**11E-15**	2.86E-12	2.02E-09	4.44E-12	2.10E-12
GO:0034109	homotypic cell-cell adhesion	**2**.**11E-15**	1.05E-12	1.34E-09	1.26E-14	1.05E-10
GO:0070486	leukocyte aggregation	**2**.**43E-15**	1.60E-12	2.40E-09	2.00E-14	1.82E-10
GO:0098602	single organism cell adhesion	**4**.**87E-15**	1.01E-12	7.14E-10	1.42E-11	7.25E-13
GO:0050776	regulation of immune response	**9**.**00E-15**	7.66E-11	4.01E-09	1.13E-12	5.59E-11

**Table 5:** The P-Values of GO terms corresponding to different methods on the DLBCL dataset.

ID	Name	SLDSF	RGNMF	GNMF	RPCA	PMD
		P-Value	P-Value	P-Value	P-Value	P-Value
GO:0006614	SRP-dependent cotranslational protein targeting to membrane	**1**.**70E-93**	4.29E-90	3.66E-91	1.94E-35	2.65E-92
GO:0006613	cotranslational protein targeting to membrane	**5**.**05E-93**	1.23E-89	1.05E-90	3.03E-35	7.62E-92
GO:0045047	protein targeting to ER	**4**.**13E-92**	9.48E-89	8.10E-90	7.19E-35	5.87E-91
GO:0072599	establishment of protein localization to endoplasmic reticulum	**3**.**07E-91**	6.65E-88	5.69E-89	1.65E-34	4.12E-90
GO:0000184	nuclear-transcribed mRNA catabolic process, nonsense-mediated decay	**1**.**30E-90**	2.72E-87	2.32E-88	2.46E-36	1.68E-89
GO:0070972	protein localization to endoplasmic reticulum	**1**.**46E-87**	2.51E-84	2.15E-85	5.78E-33	1.56E-86
GO:0006414	translational elongation	**1**.**47E-82**	1.84E-79	1.26E-80	2.02E-30	1.57E-80
GO:0006415	translational termination	**2**.**12E-81**	2.51E-78	2.16E-79	2.61E-30	2.80E-80
GO:0019080	viral gene expression	**4**.**89E-81**	5.62E-78	4.33E-79	7.12E-31	2.67E-79
GO:0044033	multi-organism metabolic process	**6**.**33E-80**	6.82E-77	5.27E-78	3.02E-30	3.40E-79

#### Test on the lung dataset

Lung cancer is the second most common cause of cancer-related death in women and the most common in men. In this paper, the lung cancer dataset presented by Bhattacharjee *et al*.^20^ was adopted in our experiments. In this dataset, there are 12600 genes in 203 samples. The 203 samples include histologically defined lung adenocarcinomas (139 samples), squamous cell lung carcinomas (21 samples), pulmonary carcinoids (20 samples), small-cell lung carcinomas cases (6 samples), and normal lung samples (17 samples).

Table 3 shows the P-Values of top 10 closely related lung cancer GO terms corresponding to the characteristic genes selected by five methods: SLDSF, RGNMF, GNMF, RPCA and PMD. In this table, ‘None’ denotes that the method cannot select genes in the GO term. SLDSF, RGNMF, GNMF and PMD can select genes in the 10 GO terms while RPCA cannot. This means that the genes selected by SLDSF, RGNMF, GNMF and PMD may have similar biological processes. In all the 10 GO terms, the SLDSF method provides much better performances than other four methods.

**Figure 3:**
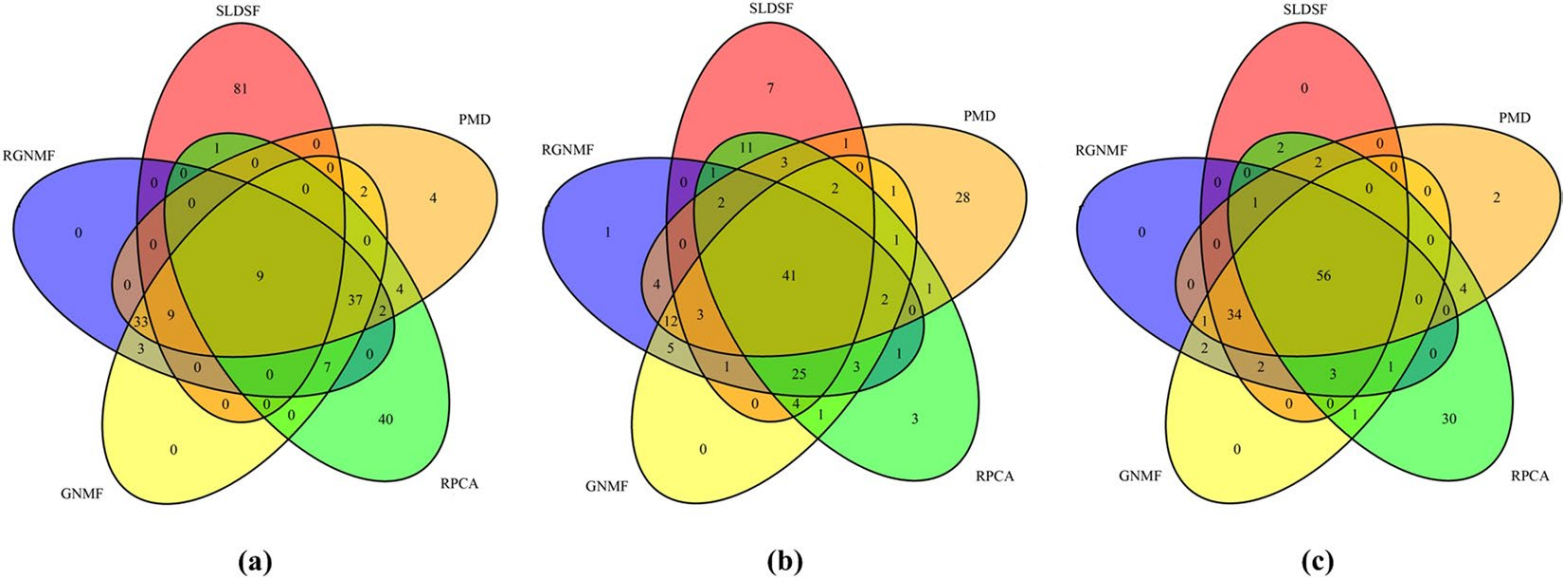
Venn diagram of genes selected by five methods on **(a)** lung cancer dataset, **(b)** leukemia dataset and **(c)** DLBCL dataset.

The genes selected by SLDSF need to be further analyzed. A Venn diagram of genes selected by five methods is shown in Fig. 3(a). We denote the ‘unique’ characteristic gene as the gene selected only by one method. From Fig. 3(a), it can be seen that there are 9 genes shared by all five methods and SLDSF can select more ‘unique’ characteristic genes (up to 81 ‘unique’ characteristic genes) than other methods. This explains why SLDSF can obtain much better performance than other methods in the GO terms in Table 3 and indicates that the 81 ‘unique’ genes are closely associated with these GO terms. The ‘unique’ characteristic genes selected by SLDSF should be further investigated to determine whether they are associated with lung cancer.

We studied the ‘unique’ genes selected by SLDSF according to the existing literature. The top 5 ‘unique’ characteristic genes selected by SLDSF are analyzed and they are shown in bold in the following explanations. For gene **GAPDH (35905_s_at)**, it was shown that the levels of GAPDH protein were significantly up-regulated in lung squamous cell carcinoma tissues by clinical tissue studies^29^. MAPK1, SRC, SMAD4, **EEF1A1 (1288_s_at)**, TRAF2 and PLCG1 might be involved in smoking-induced lung cancer by interacting with each other which indicated that they might be responsible for the development of smoking-induced lung cancer^30^. **IGHV4-31 (37864_s_at)** has been detected as a candidate gene in peripheral blood mononuclear cells (PBMC) and tumor tissue groups of non-small cell lung cancer^31^. **CYAT1 (33273_f_at)** is one of the most frequently ranked genes responsible for that clustering through the method proposed by Mondal *et al*.^32^ on the lung dataset. Czajkowski *et al*. reported perfect classification accuracy with only 3 genes: 37947_at, **33499_s_at (IGHA2)** and 36528_at on the lung cancer dataset, indicating that these 3 genes are very crucial for lung cancer^33^.

#### Test on the leukemia dataset

The leukemia dataset has already become a benchmark dataset in cancer gene selection. It consists of 11 cases of acute myelogenous leukemia and 27 cases of acute lymphoblastic leukemia^21^. The leukemia dataset is summarized by a 5000 × 38 matrix (5000 genes in 38 samples) for further study.

The P-Values of the top 10 closely related leukemia GO terms corresponding to the characteristic genes selected by five methods are shown in Table 4. From Table 4, it can be found that, for 9 GO terms, the SLDSF method outperforms RGNMF, GNMF, RPCA and PMD methods. RPCA has the lowest P-value in the term GO:0001775.

To further study the selected genes by these methods on the leukemia dataset, a Venn diagram is shown in Fig. 3(b). In Fig. 3(b), we can observe that there are 41 genes shared by all five methods. The SLDSF method can select 7 ‘unique’ characteristic genes which are neglected by the other methods.

Moreover, we verified these ‘unique’ genes according to the existing literature to determine whether these genes are associated with leukemia or not. The top 5 ‘unique’ characteristic genes selected by SLDSF are analyzed and they are shown in bold in the following explanations. **LAPTM5 (J04990_at)** decreased autophagy activity and might represent a potential target modulating autophagy activity to increase sensitivity to chemotherapy in treatment of leukemia^34^. **FOS (J04130_s_at)** has a significant function in regulating cell proliferation, cell differentiation and cell transformation in leukemia and it was detected and validated in the paper^35^. Immune-related gene **LYZ (U49835_s_at)** were highly expressed in THP1 cells in leukemia^36^. According to^37^, as a direct target of activated NOTCH1, **CCND3 (M21624_at)** is up-regulated in T-cell acute lymphoblastic leukemia. By mediating **JUNB (X60486_at)**, miRNA-149 promotes cell proliferation and inhibits apoptosis in T-cell acute lymphoblastic leukemia^38^.

#### Test on the DLBCL dataset

Diffuse large B cell lymphoma (DLBCL) is the most common lymphoid malignancy in adults. Here, we adopt the DLBCL dataset presented by Shipp *et al*.^22^. This dataset contains 7129 genes in 58 cancer samples. DLBCL study patients were divided into 2 discrete categories: 32 ‘cured’ patients and 26 ‘fatal/refractory’ patients.

Table 5 lists the P-Values of the top 10 closely related DLBCL GO terms corresponding to the characteristic genes selected by five methods. From Table 5, it can be seen that SLDSF provides better performances than that of other methods for all 10 terms.

To further study the genes selected by these methods on the DLBCL dataset, a Venn diagram is shown in Fig. 3(c). From Fig. 3(c), we can find that there are 56 genes shared by all five methods. SLDSF, RGNMF and GNMF have no ‘unique’ characteristic genes, and PMD has only 2 ‘unique’ characteristic genes. This suggests that the results of SLDSF, RGNMF, GNMF and PMD in Table 5 are very similar. There are 30 ‘unique’ characteristic genes are selected by RPCA, this may explain why RPCA has worse performance in Table 5.

### Test on RNA-Seq Datasets

The Cancer Genome Atlas (TCGA) plan attempts to apply genomic analysis techniques, especially the use of large-scale genome sequencing, to draw all human cancers genome variation map. In this section, we choose two kinds of RNA-Seq datasets, i.e., esophageal cancer (ESCA) and squamous cell carcinoma of head and neck (HNSC), which can be downloaded from TCGA (http://tcgadata.nci.nih.gov/tcga/). Here, we also adopt the 3-Layer SLDSF with 200 samples. Since RGNMF and GNMF cannot select genes in the GO terms on the two datasets, we only compared SLDSF, RPCA and PMD. The results of SLDSF, RPCA and PMD on ESCA dataset and HNSC dataset are summarized in Tables 6 and 7, respectively. In the tables, the best results among three methods are shown in bold. For simplicity, only the P-values of top 10 GO terms for each method are shown in this paper.

**Table 6:** The P-Values of GO terms corresponding to different methods on the ESCA dataset.

ID	Name	SLDSF	RPCA	PMD
		P-Value	P-Value	P-Value
GO:0042060	wound healing	**7**.**30E-16**	8.20E-13	7.56E-12
GO:0009611	response to wounding	**1**.**38E-12**	4.01E-10	4.01E-10
GO:0022610	biological adhesion	2.01E-12	**5**.**40E-14**	3.37E-13
GO:0006955	immune response	**3**.**37E-12**	9.95E-11	9.95E-11
GO:0007155	cell adhesion	9.34E-12	**2**.**71E-13**	1.63E-12
GO:0043588	skin development	**1**.**06E-11**	**1**.**06E-11**	None
GO:0007010	cytoskeleton organization	**8**.**65E-11**	1.39E-08	**8**.**65E-11**
GO:0050776	regulation of immune response	**9**.**56E-11**	6.12E-10	3.70E-09
GO:0034109	homotypic cell-cell adhesion	**1**.**92E-10**	1.59E-08	**1**.**92E-10**
GO:0098609	cell-cell adhesion	**5**.**20E-10**	3.04E-09	3.04E-09

**Table 7:** The P-Values of GO terms corresponding to different methods on the HNSC dataset.

ID	Name	SLDSF	RPCA	PMD
		P-Value	P-Value	P-Value
GO:0042060	wound healing	**9**.**46E-16**	5.38E-11	1.69E-11
GO:0031581	hemidesmosome assembly	**6**.**00E-14**	2.27E-09	None
GO:0009611	response to wounding	**1**.**80E-12**	1.09E-08	2.88E-08
GO:0022610	biological adhesion	**2**.**78E-12**	5.73E-09	9.48E-10
GO:0034330	cell junction organization	**4**.**26E-12**	5.69E-10	1.25E-07
GO:0043588	skin development	1.24E-11	7.65E-18	**7**.**50E-27**
GO:0007010	cytoskeleton organization	**1**.**88E-11**	2.56E-07	6.43E-07
GO:0034329	cell junction assembly	**3**.**16E-11**	5.69E-10	1.19E-06
GO:0045104	intermediate filament cytoskeleton organization	6.83E-11	**5**.**75E-11**	8.77E-11
GO:0007155	cell adhesion	**6**.**85E-11**	2.21E-08	7.91E-10

#### Test on the ESCA dataset

The ESCA data are the RNA-Seq data of esophageal cancer. It includes 192 samples and 20502 genes. There are 9 normal samples and 183 diseased samples.

Table 6 shows the P-Values of the top 10 closely related ESCA GO terms corresponding to the characteristic genes selected by three methods: SLDSF, RPCA and PMD. In this table, ‘None’ denotes that the method cannot select genes in the GO term. SLDSF outperforms RPCA and PMD in 5 GO terms. In GO:0043588, SLDSF has the best performance, same as RPCA. In GO:0007010 and GO:0034109, SLDSF has the best performance, same as PMD. In GO:0022610 and GO:0007155, RPCA has the lowest P-Values.

**Figure 4:**
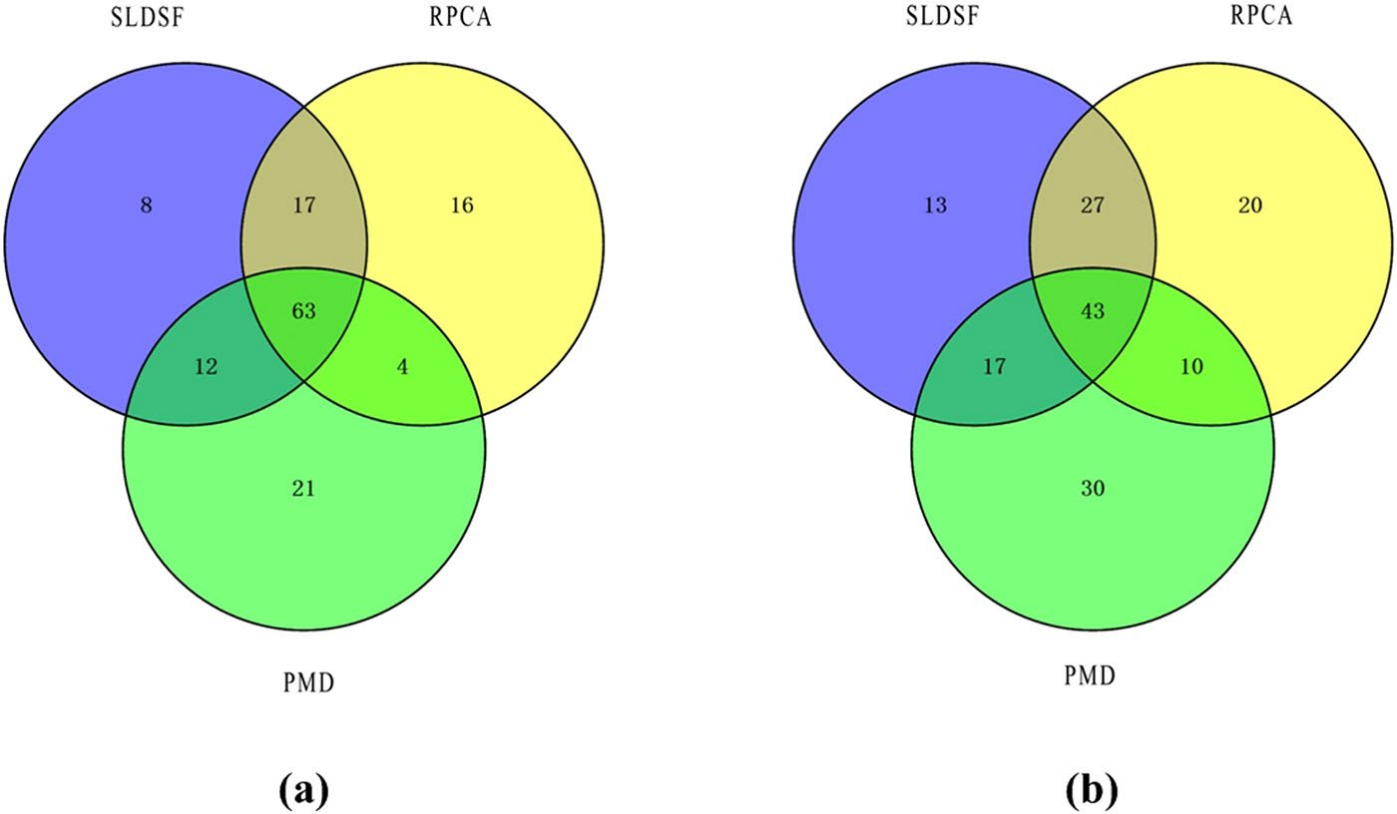
The Venn diagram of genes selected by three methods on **(a)** ESCA dataset and **(b)** HNSC dataset.

A Venn diagram of genes selected by three methods is shown in Fig. 4(a). We denote the ‘unique’ characteristic gene as the gene selected only by one method while neglected by other methods. From Fig. 4(a), there are 63 genes shared by all methods and SLDSF can select 8 ‘unique’ characteristic genes. The ‘unique’ characteristic genes should be further investigated to determine whether they are associated with ESCA.

We studied the ‘unique’ genes selected by SLDSF according to the existing literatures. The top 5 ‘unique’ characteristic genes selected by SLDSF are analyzed, and they are shown in bold in the following explanations. Shen *et al*. have performed the first GWAS (Genome-wide Association Study) of esophageal squamous cell carcinoma in the MHC (Major Histocompatibility Complex) region on the subjects from high risk areas in northern China and found three important independent susceptibility loci containing three biologically interesting candidate genes, i.e., HLA-DQA1, TRIM27 and **DPCR1**^39^. Li *et al*. found that DRD2/**PPP1R1B** (also known as DARPP-32) expression is associated with tumor progression and that DRD2/ **PPP1R1B** expression may help predict prognosis in patients with esophageal squamous cell carcinoma^40^. In^41^, **MUC17**, MUC5B and MUC6 gene mutations in tumor region T4A of esophageal squamous cell carcinoma predict the perturbation of O-glycan biosynthesis and processing. The presence of activating mutations within **EGFR** in esophageal adenocarcinomas defines a previously unrecognized subset of gastrointestinal tumors in which **EGFR** signaling may play an important, biological role^42^. According to an analysis of genes strongly up-regulated in both esophageal adenocarcinoma and Barrett’s esophagus, **REG4** might be of particular interest as an early marker for esophageal adenocarcinoma^43^.

#### Test on the HNSC dataset

The HNSC data are the RNA-Seq data of squamous cell carcinoma of head and neck. It includes 418 samples and 20502 genes. There are 20 normal samples and 398 diseased samples.

Table 7 shows the P-Values of the top 10 closely related HNSC GO terms corresponding to the characteristic genes selected by three methods. SLDSF outperforms other methods in 8 GO terms. In GO:0043588, PMD has the best performance. In GO:0045104, RPCA is a little better than SLDSF.

To further study the genes selected by these methods on HNSC dataset, a Venn diagram is shown in Fig. 4(b). There are 43 genes shared by all three methods. SLDSF can select 13 ‘unique’ characteristic genes. We verified these ‘unique’ genes according to the existing literature to determine whether these genes are associated with HNSC or not.

Here, the top 5 ‘unique’ characteristic genes selected by SLDSF are investigated. Kinoshita *et al*. demonstrated that **LAMB3** functions as an oncogene and strongly contributes to cancer cell migration and invasion in HNSC^44^. **CD44** isoforms mediate migration, proliferation, and cisplatin sensitivity in HNSC. Furthermore, expression of certain **CD44** variants may be important molecular markers for HNSC progression^45^. **HSP90AA1** and **CTSD** are down-regulated in HNSC after the combination treatment of cilengitide and cisplatin when compared to cisplatin alone^46^. **CTL1** was identified as an up-regulated gene in HNSC^47^.

### Global Cancer Genes Selected by SLDSF

We have used SLDSF to selected characteristic genes for different cancer types and subtypes. However the results of using our method for global cancer genes selection (independent of type/subtype) have not been discussed yet. These global genes may play an important role in the development of multiple cancers.

For microarray datasets, 3 global cancer genes (CD74, FTL and HLA-DRA) are selected by SLDSF from lung cancer dataset, leukemia dataset and DLBCL dataset. The functional description of these genes is as follows. The protein encoded by CD74 associates with class II major histocompatibility complex (MHC) and is an important chaperone that regulates antigen presentation for immune response. It also serves as a cell surface receptor for the cytokine macrophage migration inhibitory factor (MIF) which, when bound to the encoded protein, initiates survival pathways and cell proliferation. This protein also interacts with amyloid precursor protein (APP) and suppresses the production of amyloid beta (Abeta). FTL encodes the light subunit of the ferritin protein. Variations in ferritin subunit composition may affect the rates of iron uptake and release in different tissues. A major function of ferritin is the storage of iron in a soluble and nontoxic state. Defects in this light chain ferritin gene are associated with several neurodegenerative diseases and hyperferritinemia-cataract syndrome. HLA-DRA is one of the HLA class II alpha chain paralogues. Class II molecules are expressed in antigen presenting cells (APC: B lymphocytes, dendritic cells, macrophages).

For RNA-Seq datasets, there are 63 global cancer genes are selected by SLDSF from ESCA and HNSC datasets. This may indicate that ESCA and HNSC have many identical characteristic genes. For simplicity, the functional descriptions of 3 global genes (ACTB, COL1A1 and KRT13) are reported as follows. ACTB encodes one of six different actin proteins. Mutations in this gene cause Baraitser-Winter syndrome 1, which is characterized by intellectual disability with a distinctive facial appearance in human patients. COL1A1 encodes the pro-alpha1 chains of type I collagen. Mutations in this gene are associated with osteogenesis imperfecta types I-IV, Ehlers-Danlos syndrome type VIIA, Ehlers-Danlos syndrome Classical type, Caffey Disease and idiopathic osteoporosis. Reciprocal translocations between chromosomes 17 and 22, where this gene and the gene for platelet-derived growth factor beta are located, are associated with a particular type of skin tumor called dermatofibrosarcoma protuberans. The protein encoded by KRT13 is a member of the keratin gene family. Mutations in this gene and keratin 4 have been associated with the autosomal dominant disorder White Sponge Nevus. It is worth noting that FTL can be selected by SLDSF on all five datasets.

It would be interesting to see how SLDSF performs for selecting genes that are already well-known and validated oncogenes and/or suppressors. SLDSF can successfully select oncogenes when tested on five gene expression datasets. For example, three oncogenes: FOS, LCK, MYB are selected in the leukemia dataset. Four oncogenes: ERBB2, LCN2, EGFR and CCND1 can be selected in the ESCA dataset. SLDSF can also select suppressors from five gene expression datasets, for instance, RPL10 in the lung cancer, EGFR and ERBB2 in ESCA, and EEF1A1 in lung cancer, DLBCL, ESCA and HNSC. Note that EGFR and ERBB2 in ESCA data are both oncogenes and suppressors.

## Conclusions

Identifying cancer characteristic genes is important to understand the underlying genetics and the prognostic assessment of cancer. In this paper, we proposed a novel unsupervised characteristic gene selection method, SLDSF, based on sample learning and deep sparse filtering. Using sample learning to transform the sample space of the gene expression data, the genes can be better represented in the transformed sample space. By using sparse filtering to implement sample learning to avoid explicit modeling of the data distribution, sample learning can be achieved in a simple formulation effectively. Furthermore, for the gene expression data, we provide a detailed explanation of how sample learning satisfies three desirable characteristics of the sample distribution (population sparsity, high dispersal and lifetime sparsity) in sparse filtering. While traditional unsupervised characteristic gene selection methods do not take the deep structure into account, the proposed SLDSF explores deep sparse filtering to implement sample learning, with the advantage that multi-layers may learn more meaningful representations than a single layer.

In summary, the main contributions of this paper are described as follows:

- A deep learning structure, deep sparse filtering, is proposed for selecting cancer characteristic genes for the first time in the literature.

- We propose a novel idea, sample learning, for transforming the sample space of the gene expression data to select genes with deep learning. This enables us to better understand feature representations by the transformed sample space.

We investigated the number of layers and the number of samples in the proposed SLDSF method on five real gene expression datasets: lung cancer dataset, leukemia dataset, DLBCL dataset, ESCA dataset and HNSC dataset. The results of SLDSF were compared with four characteristic gene selection methods: RGNMF, GNMF, RPCA and PMD. Experimental studies on gene expression datasets consistently suggest that, SLDSF is more effective than other four methods for selecting cancer characteristic genes. Especially on the lung cancer dataset, the proposed SLDSF method significantly outperforms other four methods. The ‘unique’ genes selected by SLDSF are shown closely associated with the specific cancer dataset according to the current literatures. Furthermore, global cancer genes selected by SLDSF are analyzed. It is observed that SLDSF can find many oncogenes and/or suppressors from the studied five datasets.

The main limitation of this paper is its related biological explanations of the selected cancer characteristic genes. In this paper, we use GO analysis to evaluate the effectiveness of SLDSF and justify the selected genes based on the existing literature. Although GO analysis may not be a strong authentication way to validate an algorithm, it is recommended as an approach to evaluate the method in many papers^6,23^. However, the selected genes should be verified in biological experiments by biologists to find more meaningful biological explanations. In future, we will explore more on biological meanings of the selected cancer characteristic genes.

## Electronic supplementary material

Supplementary S1

Supplementary S2

Supplementary S3

Supplementary Dataset1
