# Supplementary material for: Cancer Characteristic Gene Selection via Sample Learning Based on Deep Sparse Filtering
Source: Sci Rep. 2018 May 29;8:8270. doi: 10.1038/s41598-018-26666-0 (PMC5974408; doi:10.1038/s41598-018-26666-0)
Supplement: Supplementary file 1 — Supplementary S1 [file 41598_2018_26666_MOESM1_ESM.pdf]

Supplementary S1

Cancer Characteristic Gene Selection via Sample Learning Based on Deep Sparse Filtering

Jian Liu[1], Yuhu Cheng[1], Xuesong Wang[1,*], Lin Zhang[1] & Z Jane Wang[2]

[1] School of Information and Control Engineering, China University of Mining and Technology, Xuzhou, 221116, China.

[2] Electrical and Computer Engineering Department, University of British Columbia, V6T 1Z4, Vancouver, BC, Canada.

* Corresponding author

X.S.W. E-mail address: wangxuesongcumt@163.com; Tel: +86-139-1345-5365

The detailed description of the RGNMF, GNMF, RPCA and PMD are given as follows.

The sparse method PMD, which is implemented by SVD, was used to select plants core genes responding to abiotic stresses. And the results indicated that PMD has superiority over the sparse method SPCA. RPCA is a robust method which was applied to discover differentially expressed genes on plants gene expression data and colon data. It is shown to have better performance than PMD and SPCA. GNMF is a graph regularized NMF method for data representation. And the application of GNMF in characteristic gene selection was achieved by Wang et al. Experiments on plants expression data and diffuse large B cell lymphoma dataset verify that GNMF has a better performance than PMD and SPCA. RGNMF is the extended version of GNMF by enforcing-norm minimization on error function, this permits RGNMF be robust to outliers and noises in data points. Compared with GNMF, NMFSC, PMD and SPCA on leukemia dataset, lung cancer dataset and DBLCL dataset, RGNMF has more excellent performance and is the state-of-art method.
