# Supplementary material for: Cancer Characteristic Gene Selection via Sample Learning Based on Deep Sparse Filtering
Source: Sci Rep. 2018 May 29;8:8270. doi: 10.1038/s41598-018-26666-0 (PMC5974408; doi:10.1038/s41598-018-26666-0)
Supplement: Supplementary file 2 — Supplementary S2 [file 41598_2018_26666_MOESM2_ESM.pdf]

# Supplementary S2

# Cancer Characteristic Gene Selection via Sample Learning Based on Deep Sparse Filtering

Jian Liu[1], Yuhu Cheng[1], Xuesong Wang[1,*], Lin Zhang[1] & Z Jane Wang[2]

[1] School of Information and Control Engineering, China University of Mining and Technology, Xuzhou, 221116, China.

[2] Electrical and Computer Engineering Department, University of British Columbia, V6T 1Z4, Vancouver, BC, Canada.

* Corresponding author

X.S.W. E-mail address: wangxuesongcumt@163.com; Tel: +86-139-1345-5365

**All 100 genes identified by SLDSF on five gene expression datasets.**

| Microarray Datasets | | | RNA-Seq Datasets | |
|---|---|---|---|---|
| **Lung Cancer** | **Leukemia** | **DLBCL** | **ESCA** | **HNSC** |
| AFFX-hum_alu_at | M25079_s_at | X17206_at | ACTB | KRT14 |
| 35905_s_at | X00274_at | D49824_s_at | COL1A1 | KRT6A |
| 1288_s_at | X57351_s_at | D79205_at | KRT13 | KRT5 |
| 33501_r_at | Z84721_cds2_at | HG2873-HT3017_at | KRT5 | KRT17 |
| 37864_s_at | X00437_s_at | X69150_at | KRT6A | COL1A1 |
| 33273_f_at | M13560_s_at | HG3549-HT3751_at | MALAT1 | KRT16 |
| 33499_s_at | M11147_at | X56932_at | ACTG1 | KRT6B |
| 33274_f_at | D64142_at | M17885_at | FN1 | ACTB |
| 35083_at | Y00433_at | HG1800-HT1823_at | JUP | KRT13 |
| 40887_g_at | M12886_at | M18000_at | ADAM6 | ADAM6 |
| 33500_i_at | M13792_at | M81757_at | KRT14 | GAPDH |
| 31962_at | L06797_s_at | X67247_rna1_at | MYH11 | COL3A1 |
| AFFX-HSAC07/X00351_3_at | X12671_rna1_at | HG3214-HT3391_at | LYZ | ACTG1 |
| 32466_at | V00594_s_at | D13413_rna1_s_at | OLFM4 | FN1 |
| AFFX-HSAC07/X00351_M_at | M16279_at | U14969_at | KRT17 | COL1A2 |
| 38194_s_at | AFFX-HUMRGE/M10098_5_at | D23660_at | COL3A1 | S100A9 |
| 37383_f_at | M33680_at | HG3364-HT3541_at | KRT4 | B2M |
| 31557_at | M33600_f_at | J00105_s_at | GAPDH | DSP |
| AFFX-HUMGAPDH/M33197_3_at | M21186_at | X03342_at | FLNA | KRT6C |
| 32438_at | X68277_at | M60854_at | PIGR | HLA-B |
| 2016_s_at | X78992_at | X06617_at | PGA4 | EEF1A1 |
| 676_g_at | M91036_rna1_at | U43901_rna1_s_at | COL1A2 | EEF2 |
| 31545_at | L19779_at | D14530_at | B2M | LOC96610 |
| 31385_at | S73591_at | L04483_s_at | CEACAM5 | CD74 |
| 32335_r_at | HG2917-HT3061_f_at | U14973_at | PABPC1 | ENO1 |

| | | | | |
|---|---|---|---|---|
| AFFX-HSAC07/X00351_5_at | HG2915-HT3059_f_at | M17733_at | FTL | PKM |
| 769_s_at | M19311_s_at | U14970_at | EEF1A1 | SFN |
| 201_s_at | M69043_at | L38941_at | ERBB2 | NDRG1 |
| 31444_s_at | L20941_at | X63527_at | PGC | KRT4 |
| 31957_r_at | X76223_s_at | HG4319-HT4589_at | EEF2 | PERP |
| 35278_at | U51240_at | X16064_at | CTSB | AHNAK |
| 35119_at | U14603_at | X62691_at | DSP | H19 |
| 32748_at | HG3576-HT3779_f_at | M84711_at | PLEC | FTL |
| 41237_at | X82240_rna1_at | L06499_at | LOC96610 | MYH9 |
| 256_s_at | J04456_at | U12404_at | MYH9 | TPT1 |
| 38691_s_at | J03077_s_at | X73460_at | S100A9 | RPL8 |
| 34643_at | X58529_at | X00274_at | SPRR3 | UBC |
| 428_s_at | M59807_at | U14968_at | CD74 | HLA-A |
| 34593_g_at | U23852_s_at | M14199_s_at | PGA3 | CTSB |
| 37004_at | AFFX-HUMRGE/M10098_3_at | M77232_rna1_at | LIPF | S100A8 |
| 32744_at | L19686_rna1_at | X00351_f_at | DMBT1 | PI3 |
| 31956_f_at | Z15115_at | AFFX-HUMGAPDH/M33197_3_at | H19 | JUP |
| 31527_at | X17042_at | M24194_at | LCN2 | FLNA |
| 37039_at | Y00787_s_at | M17886_at | HSP90AB1 | ANXA1 |
| 31505_at | AFFX-HUMRGE/M10098_M_at | M11147_at | UBC | PABPC1 |
| 327_f_at | V01512_rna1_at | U09953_at | REG1A | LAMC2 |
| 32276_at | X13546_rna1_at | AFFX-HSAC07/X00351_M_at | KRT19 | LYZ |
| 32318_s_at | M24485_s_at | U14972_at | TPT1 | SPARC |
| 1367_f_at | M63438_s_at | X15940_at | DPCR1 | S100A2 |
| 32334_f_at | J04164_at | X03689_s_at | HSPB1 | TNC |
| 347_s_at | X03934_at | L19527_at | KRT16 | KRT1 |
| 41827_f_at | X67951_at | X80822_at | PKM | SPRR3 |
| 34645_at | X13794_rna1_at | Z28407_at | HLA-B | ALDOA |

| | | | | |
|---|---|---|---|---|
| AFFX-HUMGAPDH/M33197_M_at | HG2279-HT2375_at | S79522_at | AHNAK | MUC7 |
| 32412_at | M96326_rna1_at | Z12962_at | PPP1R1B | PKP1 |
| 41164_at | X59417_at | M13934_cds2_at | CD24 | HLA-C |
| AFFX-HUMGAPDH/M33197_5_at | Z23090_at | AFFX-HSAC07/X00351_3_at | HSP90AA1 | KRT19 |
| 31697_s_at | X17093_at | Z26876_at | S100A8 | COL17A1 |
| 31568_at | M34516_at | U06155_s_at | ANXA1 | HSPB1 |
| 34608_at | M62831_at | hum_alu_at | NDRG1 | HSP90AB1 |
| 38061_at | HG1612-HT1612_at | X55954_at | PERP | HSPA1A |
| 41745_at | M21388_r_at | X55715_at | FTH1 | YWHAZ |
| 31330_at | D30655_at | D78361_at | TNC | KRT15 |
| 34085_at | AFFX-M27830_M_at | J04617_s_at | KRT8 | PSAP |
| 33994_g_at | U22376_cds2_s_at | HG33-HT33_at | YWHAZ | RPL3 |
| 31538_at | M23323_s_at | Z49148_s_at | KRT6B | RPS6 |
| 31722_at | M37033_at | HG821-HT821_at | ALDOA | PLEC |
| 33116_f_at | D00749_s_at | L11566_at | MUC6 | LTF |
| 34105_f_at | X70683_at | M64716_at | PKP1 | SLC2A1 |
| 33676_at | M92287_at | HG311-HT311_at | ACTN4 | HSP90AA1 |
| 40886_at | U52101_at | X98482_r_at | MUC17 | BPIFB1 |
| 1315_at | M33764_at | AB002533_at | HSPA8 | TUBA1B |
| 32330_at | M11722_at | M55409_s_at | SRRM2 | MYH2 |
| 36785_at | U05259_rna1_at | M13560_s_at | ATP1A1 | HSPA8 |
| 1366_i_at | X03100_cds2_at | M10277_s_at | LDHA | LDHA |
| 39916_r_at | X14046_at | U49869_rna1_at | EGFR | SDC1 |
| 33667_at | X51521_at | M36072_at | RPL8 | MUC5B |
| 34160_at | HG1153-HT1153_at | M31520_at | SPRR1B | TUBB |
| 32435_at | M57466_s_at | HG613-HT613_at | TUBB | SPRR1B |
| 34592_at | HG1980-HT2023_at | X01677_f_at | TMSB4XP8 | TGFBI |
| 38126_at | J03779_at | HG4542-HT4947_at | EIF4G2 | DSG3 |
| 32341_f_at | U01317_cds4_at | X53777_at | RPS3 | ATP1A1 |

| | | | | |
|---|---|---|---|---|
| 35016_at | D88270_at | L20688_at | AGR2 | CALR |
| 31511_at | X65965_s_at | U12465_at | HLA-C | KRT10 |
| 33674_at | HG688-HT688_f_at | HG2815-HT4023_s_at | PSAP | ANXA2 |
| 39830_at | U73824_at | U14971_at | EIF1 | GSTP1 |
| 2035_s_at | AFFX-M27830_5_at | X79234_at | ENO1 | ZG16B |
| 37449_i_at | M19507_at | Z70759_at | C3 | GNB2L1 |
| 31951_s_at | J04990_at | AFFX-HUMGAPDH/M33197_5_at | HLA-A | LAMB3 |
| 32436_at | U50743_at | X56997_rna1_at | TUBA1B | ITGA6 |
| 32315_at | U49835_s_at | Z19554_s_at | GNAS | TMSB10 |
| 33659_at | M87789_s_at | U58682_at | CLDN18 | CD44 |
| 34091_s_at | HG1862-HT1897_at | AFFX-HSAC07/X00351_5_at | PI3 | CFL1 |
| 33656_at | S53911_at | M33600_f_at | S100A7 | P4HB |
| 41178_at | M21624_at | HG2815-HT2931_at | REG4 | RPLP1 |
| 39248_at | M86667_at | D32129_f_at | SERPINA1 | RPS4X |
| 31509_at | M92843_s_at | V00594_s_at | ITGB4 | ACTA1 |
| 34644_at | L09604_at | AFFX-HUMGAPDH/M33197_M_at | CCND1 | CTSD |
| 36795_at | X04500_at | HG3576-HT3779_f_at | SPTBN1 | GJB2 |
| 32437_at | X03068_f_at | X52851_rna1_at | CD44 | TTN |
