# Supplementary material for: Cancer Characteristic Gene Selection via Sample Learning Based on Deep Sparse Filtering
Source: Sci Rep. 2018 May 29;8:8270. doi: 10.1038/s41598-018-26666-0 (PMC5974408; doi:10.1038/s41598-018-26666-0)
Supplement: Supplementary file 3 — Supplementary S3 [file 41598_2018_26666_MOESM3_ESM.pdf]

Supplementary S3

Cancer Characteristic Gene Selection via Sample Learning Based on Deep Sparse Filtering

Jian Liu[1], Yuhu Cheng[1], Xuesong Wang[1,*], Lin Zhang[1] & Z Jane Wang[2]

[1] School of Information and Control Engineering, China University of Mining and Technology, Xuzhou, 221116, China.

[2] Electrical and Computer Engineering Department, University of British Columbia, V6T 1Z4, Vancouver, BC, Canada.

* Corresponding author

X.S.W. E-mail address: wangxuesongcumt@163.com; Tel: +86-139-1345-5365

We tested the influence of number of layers and samples in SLDSF method.

Test on Lung Cancer Dataset

Table S1 The P-Values of GO terms corresponding to different number of layers of SLDSF on lung cancer dataset.

ID	Name	1-Layer	2-Layer	3-Layer	4-Layer	5-Layer
		P-Value	P-Value	P-Value	P-Value	P-Value
GO:0000184	nuclear-transcribed mRNA catabolic process, nonsense-mediated decay	3.70E-54	6.96E-56	**5.05E-72**	1.912E-67	8.47E-68
GO:0006614	SRP-dependent cotranslational protein targeting to membrane	5.41E-56	8.71E-58	**7.03E-72**	1.05E-69	1.54E-67
GO:0006613	cotranslational protein targeting to membrane	1.08E-55	1.78E-57	**1.69E-71**	2.46E-69	3.49E-67
GO:0045047	protein targeting to ER	4.08E-55	7.08E-57	**9.22E-71**	1.27E-68	1.71E-66
GO:0072599	establishment of protein localization to endoplasmic reticulum	1.47E-54	2.67E-56	**4.68E-70**	6.13E-68	7.84E-66
GO:0070972	protein localization to endoplasmic reticulum	3.4E-52	7.54E-54	**4.61E-67**	4.88E-65	5.06E-63
GO:0019080	viral gene expression	2.31E-50	8.20E-52	5.18E-64	**2.12E-64**	1.46E-62
GO:0044033	multi-organism metabolic process	1.36E-49	5.12E-51	4.62E-63	**1.89E-63**	1.23E-61
GO:0019083	viral transcription	3.62E-49	1.21E-50	**6.96E-63**	4.92E-61	3.42E-59
GO:0006415	translational termination	3.62E-48	1.12E-49	**5.27E-62**	3.94E-60	2.90E-58
GO:0000956	nuclear-transcribed mRNA catabolic process	2.05E-46	7.26E-48	**5.36E-62**	5.27E-58	2.35E-58

Table S2 The P-Values of GO terms corresponding to different number of samples needs to be learned of SLDSF on lung cancer dataset.

ID	Name	50-Samples	100-Samples	200-Samples	500-Samples	1000-Samples
		P-Value	P-Value	P-Value	P-Value	P-Value
GO:0000184	nuclear-transcribed mRNA catabolic process, nonsense-mediated decay	7.50E-49	2.84E-63	**5.05E-72**	1.91E-67	1.05E-69
GO:0006614	SRP-dependent cotranslational protein targeting to membrane	1.50E-50	6.41E-63	**7.03E-72**	1.05E-69	2.46E-69
GO:0006613	cotranslational protein targeting to membrane	2.84E-50	1.38E-62	**1.69E-71**	2.46E-69	1.27E-68
GO:0045047	protein targeting to ER	9.75E-50	6.09E-62	**9.22E-71**	1.27E-68	6.13E-68
GO:0072599	establishment of protein localization to endoplasmic reticulum	3.18E-49	2.53E-61	**4.68E-70**	6.13E-68	6.61E-70
GO:0070972	protein localization to endoplasmic reticulum	4.96E-47	1.08E-58	**4.61E-67**	4.88E-65	4.88E-65
GO:0019080	viral gene expression	1.97E-45	2.26E-56	**5.18E-64**	3.52E-62	3.52E-62
GO:0044033	multi-organism metabolic process	1.03E-44	1.59E-55	**4.62E-63**	2.96E-61	2.96E-61
GO:0019083	viral transcription	2.85E-44	3.42E-55	**6.96E-63**	4.92E-61	4.92E-61
GO:0006415	translational termination	2.73E-43	3.12E-54	**5.27E-62**	3.94E-60	3.94E-60
GO:0000956	nuclear-transcribed mRNA catabolic process	1.16E-41	2.09E-54	**5.36E-62**	5.273E-58	3.58E-60

SLDSF is a deep structure for sample learning. Therefore, the influence of number of layers and samples should be studied. Firstly, we tested the number of layers and the results were summarized in Table S1. Here, the number of samples was set to 200. Table S1 shows the P-Values of the top 10 closely related lung cancer GO terms corresponding to the characteristic genes selected by different layers. There are 11 GO terms that are non-overlapped. From Table S1, we can note that in 9 GO terms the 3-Layer SLDSF method achieved the best performance than the other number of layers. Only in the GO terms: GO:0019080 and GO:0044033, 4-Layer SLDSF had a slightly better results than 3-Layer SLDSF. Among all the number of layers, 1-Layer SLDSF, which is also considered as SLSF method, achieved the worst performance. With the increase in the number of layers, the performance is better. This observation supports that the deep structure of SLDSF can learn more meaningful representations and is very effective for selecting characteristic genes. However, 4-Layer SLDSF and 5-Layer SLDSF

yield worse performances than 3-Layer SLDSF. The main reason may be that, for this specific dataset too many layers (more than 3 layers) may lead to overfitting. In other words, we should determine a reasonable number of layers.

Then, the number of samples needs to be learned was tested. Here, we used the 3-Layer SLDSF method to perform the experiments. Table S2 shows the P-Values of the top 10 closely related lung cancer GO terms corresponding to the characteristic genes selected by different samples. There are 11 GO terms that are non-overlapped. Among 50, 100 and 200 samples, with the increase of the number of samples, the performance was generally better. SLDSF can achieve the best performance in all 11 GO terms when the number of samples is 200. While the number of samples reaches 500 and 1000, the results become saturated. This indicates that sample learning using the SLDSF method is valid for selecting characteristic genes.

Test on Leukemia Dataset

Table S3 The P-Values of GO terms corresponding to different number of layers of SLDSF on leukemia dataset.

ID	Name	1-Layer	2-Layer	3-Layer	4-Layer	5-Layer
		P-Value	P-Value	P-Value	P-Value	P-Value
GO:0006955	immune response	2.30E-14	8.86E-15	**2.69E-18**	6.68E-16	3.27E-15
GO:0001775	cell activation	1.14E-12	5.74E-13	**8.94E-18**	8.82E-16	3.09E-15
GO:0045321	leukocyte activation	3.70E-12	1.59E-10	**2.28E-16**	4.24E-13	1.11E-12
GO:0007159	leukocyte cell-cell adhesion	1.44E-13	1.31E-10	**5.86E-16**	3.44E-12	4.73E-14
GO:0046649	lymphocyte activation	1.62E-11	7.90E-10	**8.59E-16**	2.34E-12	5.53E-12
GO:0016337	single organismal cell-cell adhesion	1.77E-12	6.78E-10	**1.11E-15**	2.05E-11	5.28E-13
GO:0034109	homotypic cell-cell adhesion	4.50E-13	3.36E-10	**2.11E-15**	9.53E-12	1.49E-13
GO:0070486	leukocyte aggregation	5.85E-13	5.37E-10	**2.43E-15**	1.56E-11	2.06E-13
GO:0098602	single organism cell adhesion	6.91E-13	1.96E-09	**4.87E-15**	6.46E-11	1.90E-12
GO:0050776	regulation of immune response	4.47E-10	1.76E-09	**9.00E-15**	7.79E-13	1.41E-10
GO:0002684	positive regulation of immune system process	2.82E-12	7.05E-10	**2.35E-13**	2.41E-12	5.32E-11
GO:0022610	biological adhesion	8.95E-12	6.17E-09	**8.78E-13**	1.25E-09	1.35E-11
GO:0000302	response to reactive oxygen species	1.31E-10	**5.21E-12**	6.27E-12	3.86E-11	6.62E-11
GO:0006952	defense response	7.47E-09	1.29E-10	1.89E-10	1.05E-10	**5.86E-11**
GO:0010035	response to inorganic substance	**9.94E-11**	6.70E-10	5.10E-09	2.40E-09	4.40E-09
GO:0051251	positive regulation of lymphocyte activation	1.14E-11	1.61E-09	**3.24E-14**	2.71E-12	8.04E-11
GO:0050778	positive regulation of immune response	2.09E-10	1.10E-09	**1.80E-13**	3.40E-12	7.69E-11

Table S4 The P-Values of GO terms corresponding to different number of samples needs to be learned of SLDSF on leukemia dataset.

ID	Name	50-Samples	100-Samples	200-Samples	500-Samples	1000-Samples
		P-Value	P-Value	P-Value	P-Value	P-Value
GO:0006955	immune response	3.85E-16	1.43E-17	**2.69E-18**	8.86E-15	1.29E-16
GO:0001775	cell activation	3.05E-14	6.84E-15	**8.94E-18**	6.83E-16	6.42E-17
GO:0045321	leukocyte activation	1.05E-11	2.11E-13	**2.28E-16**	2.11E-13	2.03E-14
GO:0007159	leukocyte cell-cell adhesion	6.15E-13	1.21E-11	**5.86E-16**	1.04E-12	1.04E-12
GO:0046649	lymphocyte activation	5.41E-11	8.73E-14	**8.59E-16**	8.73E-14	8.73E-14
GO:0016337	single organismal cell-cell adhesion	5.21E-12	9.27E-12	**1.11E-15**	9.77E-13	9.77E-13
GO:0034109	homotypic cell-cell adhesion	1.81E-12	3.33E-11	**2.11E-15**	3.06E-12	3.06E-12
GO:0070486	leukocyte aggregation	2.66E-12	5.04E-11	**2.43E-15**	4.37E-12	4.37E-12
GO:0098602	single organism cell adhesion	1.74E-11	3.08E-11	**4.87E-15**	3.49E-12	3.49E-12
GO:0050776	regulation of immune response	1.84E-11	4.36E-12	**9.00E-15**	4.36E-12	3.43E-11
GO:0022610	biological adhesion	1.35E-11	1.82E-10	**8.78E-13**	1.82E-10	4.21E-12
GO:0002684	positive regulation of immune system process	6.54E-12	1.23E-11	**2.35E-13**	1.23E-11	1.23E-11
GO:0050778	positive regulation of immune response	7.69E-11	1.40E-12	**1.80E-13**	1.40E-12	1.39E-11
GO:0051251	positive regulation of lymphocyte activation	8.04E-11	7.69E-12	**3.24E-14**	7.69E-12	1.17E-10

First of all, we tested the number of layers and samples needs to be learned of SLDSF method. Here, the number of samples was set to 200. Table S3 shows the P-Values of the top 10 closely related leukemia cancer GO terms corresponding to the characteristic genes selected by different layers. There are 17 GO terms that are non-overlapped. From Table S3 we can note that in 14 GO terms 3-Layer SLDSF method can achieve the best performance. In the GO term: GO:0010035, 1-Layer SLDSF has the best result. 2-Layer SLDSF obtains the best performance in the GO term: GO:0000302. 5-Layer SLDSF achieves the best performance in the GO term: GO:0006952. On the whole, 3-Layer SLDSF has better results than the other cases. Moreover, the number of samples needs to be learned was tested. Here, we used 3-Layer SLDSF method. Table S4 shows the P-Values of the top 10 closely related leukemia cancer GO terms corresponding to the characteristic genes selected by different samples. There are 14 GO terms that are non-overlapped. When the number of samples is 200, SLDSF can achieve the best performance in all 10 GO terms.

Test on DLBCL Dataset

Table S5 The P-Values of GO terms corresponding to different number of samples needs to be learned of SLDSF on DLBCL dataset.

ID	Name	1-Layer	2-Layer	3-Layer	4-Layer	5-Layer
		P-Value	P-Value	P-Value	P-Value	P-Value
GO:0006614	SRP-dependent cotranslational protein targeting to membrane	1.28E-90	2.15E-91	**1.70E-93**	7.69E-89	1.28E-90
GO:0006613	cotranslational protein targeting to membrane	3.67E-90	6.39E-91	**5.05E-93**	2.15E-88	3.67E-90
GO:0045047	protein targeting to ER	2.82E-89	5.21E-90	**4.13E-92**	1.56E-87	2.82E-89
GO:0072599	establishment of protein localization to endoplasmic reticulum	1.98E-88	3.87E-89	**3.07E-91**	1.03E-86	1.98E-88
GO:0000184	nuclear-transcribed mRNA catabolic process, nonsense-mediated decay	8.10E-88	1.64E-88	**1.30E-90**	4.06E-86	8.10E-88
GO:0070972	protein localization to endoplasmic reticulum	7.49E-85	1.84E-85	**1.46E-87**	3.11E-83	7.49E-85
GO:0006414	translational elongation	4.93E-80	2.80E-80	**1.47E-82**	1.08E-78	4.93E-80
GO:0006415	translational termination	7.49E-79	2.64E-79	**2.12E-81**	2.17E-77	7.49E-79
GO:0019080	viral gene expression	1.59E-78	7.48E-79	**4.89E-81**	3.80E-77	1.59E-78
GO:0044033	multi-organism metabolic process	1.94E-77	9.66E-78	**6.33E-80**	4.34E-76	1.94E-77

Table S6 The P-Values of GO terms corresponding to different number of samples needs to be learned of SLDSF on DLBCL dataset.

ID	Name	50-Samples	100-Samples	200-Samples	500-Samples	1000-Samples
		P-Value	P-Value	P-Value	P-Value	P-Value
GO:0006614	SRP-dependent cotranslational protein targeting to membrane	2.78E-87	2.15E-91	**1.70E-93**	4.35E-89	8.59E-87
GO:0006613	cotranslational protein targeting to membrane	7.76E-87	6.39E-91	**5.05E-93**	1.25E-88	2.39E-86
GO:0045047	protein targeting to ER	5.63E-86	5.21E-90	**4.13E-92**	9.61E-88	1.74E-85
GO:0072599	establishment of protein localization to endoplasmic reticulum	3.74E-85	3.87E-89	**3.07E-91**	6.74E-87	1.15E-84
GO:0000184	nuclear-transcribed mRNA catabolic process, nonsense-mediated decay	1.47E-84	1.64E-88	**1.30E-90**	2.75E-86	4.52E-84
GO:0070972	protein localization to endoplasmic reticulum	1.12E-81	1.84E-85	**1.46E-87**	2.54E-83	3.45E-81
GO:0006414	translational elongation	5.35E-77	2.80E-80	**1.47E-82**	2.27E-78	1.81E-76
GO:0006415	translational termination	7.74E-76	2.64E-79	**2.12E-81**	2.52E-77	2.38E-75
GO:0019080	viral gene expression	1.59E-75	7.48E-79	**4.89E-81**	6.24E-77	5.13E-75
GO:0044033	multi-organism metabolic process	1.82E-74	9.66E-78	**6.33E-80**	7.57E-76	5.86E-74

We tested the number of layers and samples needed to be learned in SLDSF on the DLBCL dataset. Here, the number of samples was set to 200. Table S5 shows the P-Values of the top 10 closely related DLBCL GO terms corresponding to the characteristic genes selected by different samples. There are 10

GO terms that are non-overlapped. In Table S5, we can note that in all 10 GO terms the 3-Layer SLDSF method achieved the best performance. Furthermore, the number of samples needs to be learned was tested. Here, the 3-Layer SLDSF method was used. Table S6 shows the P-Values of the top 10 closely related DLBCL GO terms corresponding to the characteristic genes selected by different samples. There are 10 GO terms that are non-overlapped. When the number of samples is set to 200, SLDSF can achieve the best performance in all 10 GO terms.
